# The respiratory health of urban indigenous children aged less than 5 years: study protocol for a prospective cohort study

**DOI:** 10.1186/s12887-015-0375-y

**Published:** 2015-05-14

**Authors:** Kerry K. Hall, Anne B. Chang, Theo P. Sloots, Jennie Anderson, Anita Kemp, Jan Hammill, Michael Otim, Kerry-Ann F. O’Grady

**Affiliations:** Queensland Children’s Medical Research Institute, Queensland University of Technology, Herston, QLD Australia; Menzies School of Health Research, Charles Darwin University, Tiwi, NT Australia; Queensland Children’s Respiratory Centre, Royal Children’s Hospital, Brisbane, QLD Australia; Sir Albert Sakzewski Virus Research Centre, Queensland Paediatric Infectious Diseases Laboratory, Royal Children’s Hospital, Brisbane, Australia; Child Health Research Centre, The University of Queensland, Herston, QLD Australia; Murri Medical, Caboolture, QLD Australia; University of Queensland Centre for Clinical Research, The University of Queensland, Herston, QLD Australia; School of Allied Health, Australian Catholic University, North Sydney, NSW Australia

**Keywords:** Acute Respiratory Illness, Urban, Economics, Primary health care centre, Aboriginal and Torres Strait Islander, Children

## Abstract

**Background:**

Despite the burden of acute respiratory illnesses (ARI) among Aboriginal and Torres Strait Islander children being a substantial cause of childhood morbidity and associated costs to families, communities and the health system, data on disease burden in urban children are lacking. Consequently evidence-based decision-making, data management guidelines, health resourcing for primary health care services and prevention strategies are lacking. This study aims to comprehensively describe the epidemiology, impact and outcomes of ARI in urban Aboriginal and Torres Strait Islander children (hereafter referred to as Indigenous) in the greater Brisbane area.

**Methods/Design:**

An ongoing prospective cohort study of Indigenous children aged less than five years registered with a primary health care service in Northern Brisbane, Queensland, Australia. Children are recruited at time of presentation to the service for any reason. Demographic, epidemiological, risk factor, microbiological, economic and clinical data are collected at enrolment. Enrolled children are followed for 12 months during which time ARI events, changes in child characteristics over time and monthly nasal swabs are collected. Children who develop an ARI with cough as a symptom during the study period are more intensely followed-up for 28 (±3) days including weekly nasal swabs and parent completed cough diary cards. Children with persistent cough at day 28 post-ARI are reviewed by a paediatrician.

**Discussion:**

Our study will be one of the first to comprehensively evaluate the natural history, epidemiology, aetiology, economic impact and outcomes of ARIs in this population. The results will inform studies for the development of evidence-based guidelines to improve the early detection, prevention and management of chronic cough and setting of priorities in children during and after ARI.

**Trial registration:**

Australia New Zealand Clinical Trial Registry Registration Number: 12614001214628. Registered 18 November 2014

## Background

Respiratory illnesses (RI) in Aboriginal and Torres Strait Islander people (here forth referred to as Indigenous) are common, serious and important. Nationally, diseases of the respiratory system, although ranked 4^th^ as the cause of death in Indigenous infants, are the commonest cause of preventable deaths [1]. Respiratory disease is the second most common reason for hospitalisation among Indigenous Australians (after renal dialysis) [1]. Lower acute RIs (ARI) account for the greatest number of hospitalisations in young Indigenous children aged under-5 years [1]. Also, the prevalence of serious chronic RIs such as non-cystic fibrosis (CF) bronchiectasis is high (one in 68 children) in Indigenous children living in remote regions [2] and this is associated with repeated episodes of hospitalised RIs [3]. Thus, Indigenous children bear a disproportionate burden of acute and chronic lower RIs [4, 5].

Worldwide, many factors have been identified as increasing the risk of developing ARI. Most of these predictors are inherently related to both the prenatal and antenatal periods and the first five years of life. These include overcrowding, malnutrition, exposure to tobacco smoke, young maternal age, low birth weight, anaemia, poverty, illiteracy, overcrowding, parental smoking, pollution, socio economic status, social behaviours, cultural factors and family history [6–9]. Despite the burden of ARI in Australian Indigenous children, there are limited studies that have examined these factors at the community level.

To date the focus on ARI in Australian Indigenous children has been almost entirely on children living in rural and remote regions of Australia, with no community-based data on disease and its social and economic impacts in their urban contemporaries. This is despite the fact that more Indigenous children live in urban Australia and socio-economic and health indices are consistently lower for urban Indigenous communities when compared to non-Indigenous populations [1]. Further, there are few studies that have examined differences in ARI incidence and predictors between urban Indigenous and non-Indigenous children at the community level, particularly in communities that are socio-economically and geographically similar. A community-based Australian study in west Sydney estimated that the reported rate of pneumonia in non-Indigenous children was 7.5/1000, compared to 12/1000 in Indigenous Children [10]. A survey in the Australian Capital Territory reported that Indigenous children had a higher prevalence of recent wheeze (21 %), wheeze with colds (36 %), dry cough at night (27 %) and parent-reported asthma (24 %) compared to 15 %, 23 %, 19 % and 15 % respectively in non- Indigenous Islander children [11]. Maternal factors and the external environment have been linked to infertility, early pregnancy loss, premature labour and low birth-weight. Children with low birth weight are six times more likely to be hospitalised with ARI than children with normal birth weight [12, 13].

In addition to the above, ARIs are also associated with social and economic costs. Those relevant to the urban Indigenous community are unknown. In non-Indigenous communities the only the Australian studies to investigate the cost associated with ARI have been conducted in cohorts of Melbourne pre-school aged children [14, 15]. In the first study of children aged 12 – 71 months that focussed on influenza like illness, the average cost of community-managed episodes (without hospitalisation) was $241 (95 % CI $191 - $291). The key cost drivers were carer time away from usual activities caring for the ill child (70 % of costs), use of non-prescription medications (5.4 %), and general practice visits (5.0 %). The patient and family met 87 % of total costs. In a further study focussing on viral respiratory illnesses, the mean cost of ARIs was AU$309 (95 % CI $263 to $354). Influenza illnesses had a mean cost of AU$904 and RSV, AU$304. These studies were however conducted in a population with relatively high social and economic indices. Data was not collected on the impact of these illnesses on health service providers themselves.

The lack of data on urban Indigenous populations has been identified as one important barrier to Closing the Gap initiatives [16]. Although more than half of Australia’s Aboriginal and Torres Strait Islander population live in urban and regional centres, most research and commentaries address the health and social issues of remote communities [17]. In the absence of such data, we plan to conduct a study that addresses some of these gaps. Here, we present our protocol for a longitudinal, community-based, cohort study of ARI in urban children aged < 5 years presenting to a presenting to a primary health care centre.

### Aims and objectives

This study aims to comprehensively describe the epidemiology, aetiology, social and economic impact and outcomes of ARI in children aged less than 5 years registered with an urban primary health care service. Our primary objectives are to determine the incidence and predictors of ARI amongst urban Indigenous children over 12 months of child observation. Our secondary objectives are to: a) determine the prevalence of chronic cough (≥4 weeks duration) following an ARI; b) determine the direct and indirect economic costs of ARI, and; c) examine nasal carriage of respiratory viruses and bacteria over a 12 month period.

## Methods/Design

### Setting

This study is being undertaken at Murri Medical (MM), a not-for-profit, Aboriginal owned and operated comprehensive primary health care service in Caboolture, a northern suburb of Brisbane, Queensland, Australia. MM provides services to the Moreton Bay Regional Council Area, a region with a comparatively lower socio-economic status than inner Brisbane areas [18], and has approximately 9500 registered clients of which approximately two-thirds identify as Aboriginal and/or Torres Strait Islander.

### Research team

The research team responsible for inception, implementation, actioning and management of the protocol includes paediatric specialists in the field of respiratory medicine, microbiology, epidemiology, nursing, health economics and biostatistics. Study specific research officers (Aboriginal women) undertake recruitment, data collection, complete participant follow-up, data entry and attend to daily study requirements such as booking of specialist reviews. A paediatrician is responsible for the review and assessment of children who develop chronic cough following an ARI.

### Study design

An ongoing prospective cohort study of Indigenous children aged less than 5 years registered with MM with 12 months of observation per child. Children who develop cough as a symptom at any time over the 12 months are subsequently followed weekly for four weeks to ascertain costs and cough outcomes. Participation in the study has no bearing on the medical care provided to children at MM, which is conducted separately and in accordance with clinic policies and procedures. Parents are also encouraged to practice normal healthcare seeking behaviours throughout the duration of their participation.

Our primary endpoint is an ARI defined as an acute illness (i.e. less than 14 days duration) with cough as symptom with or without any accompanying symptoms. Our secondary endpoints are: a) chronic cough defined as cough duration of greater than 28 days with no cessation of cough lasting more than 3 days; b) direct and indirect costs of illness in Australian dollars, and c) detection of per protocol respiratory viruses and bacteria by polymerase chain reaction (PCR) on nasal swabs.

The study has been approved by Queensland Children’s Health Services Human Research Ethics Committee (HREC/12/QRCH/169), the Medical Research Ethics Committee of the University of Queensland (2012001395) and the Human Research Ethics Committee of the Queensland University of Technology (1300000741).

### Recruitment

Recruitment began in February 2013 and is planned to continue until June 2015 to account for annual variation in ARI incidence and aetiology. All children aged less than 5 years presenting to MM during business hours (Monday to Friday, 0830 – 1700 h) are recorded on a detailed screening log by the research assistant. This log contains de-identified demographic data as well as the reason for non-participation in the study (e.g.; ineligible, refused). Parents/guardians of all children are approached by an Aboriginal researcher at the time of presentation to the clinic for assessment of eligibility. Using a plain language statement or flip chart that explains the study in detail, written informed consent is obtained from a parent(s) or carer if they agree to participate.

### Inclusion criteria

Children are eligible for the study if: a) they identify as Aboriginal and/or Torres Strait Islander; b) they are aged less than five years at time of enrolment; c) they are registered as a client of MM; d) the parent/guardian has provided written informed consent, and; e) the parent/guardian is willing and able to complete the protocol requirements.

### Exclusion criteria

Children are excluded from the study if they are planning to move from the study area in the following 12 months.

### Study participation

**Fig. 1:**
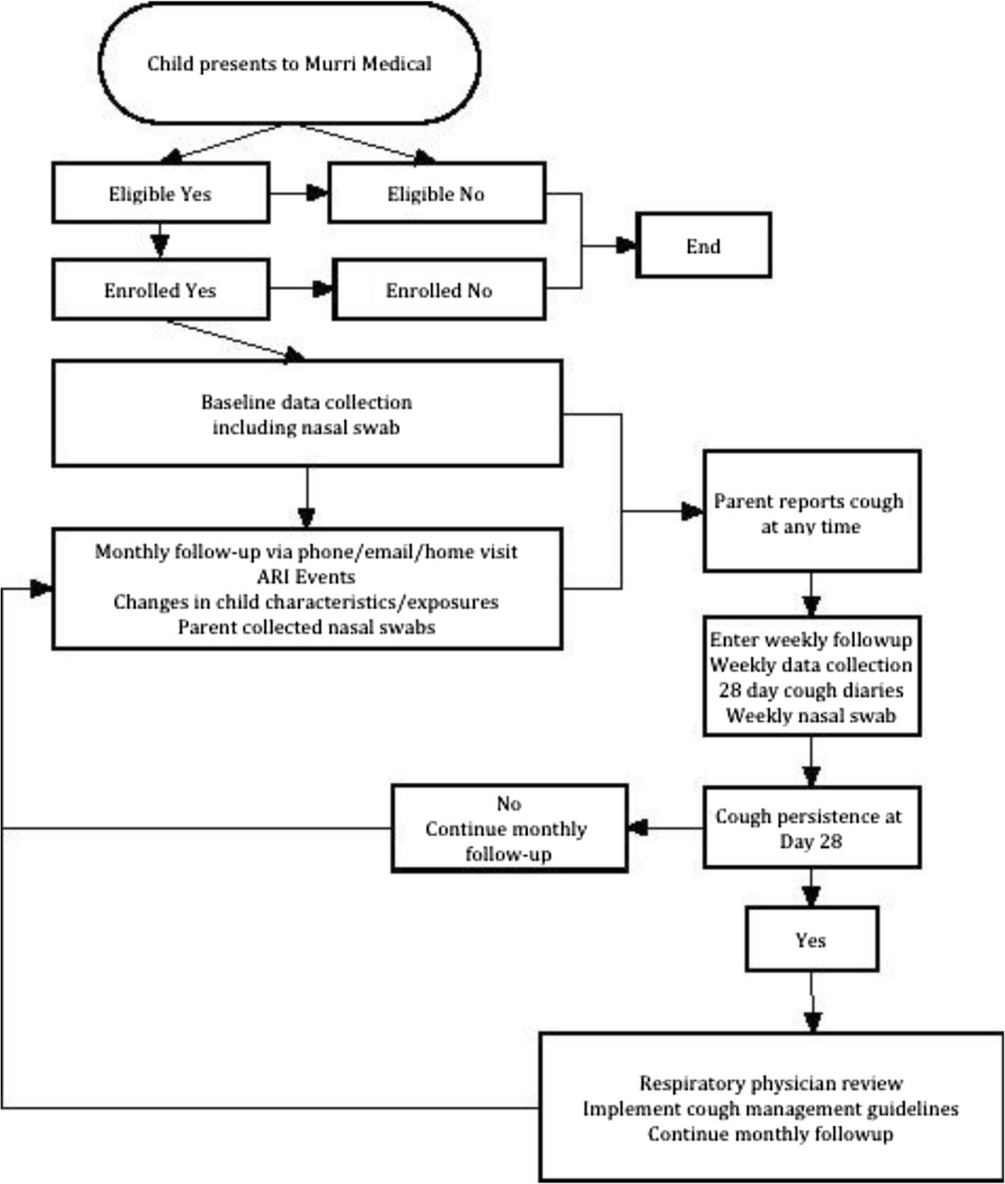
Study design

At enrolment, a dedicated Indigenous research officer completes a comprehensive questionnaire with the child and their guardian. (Fig. 1) Parent(s) or carers of enrolled children are contacted monthly via telephone, email or home visiting for 12 months. Data collection during these time points include parent-collected nasal swabs, ARI events and changes in child characteristics over time.

Children with an ARI at any time during the study (at presentation or later development of an ARI) are then intensively followed via weekly follow-up via telephone and/or email contacts for four weeks including nasal swabs, and parent-completed cough diary cards. These contacts are used to ascertain parent reported cough persistence and type, parental absence from work due to their child’s cough, missed day-care/school due to cough and whether or not the child’s cough has ceased for a period of greater than three consecutive days during the follow-up period. If a child has a persistent cough at day 28, he/she is reviewed by a paediatrician with expertise in respiratory medicine at the Queensland Children’s Respiratory Centre in Brisbane. At this review a comprehensive assessment is completed in accordance with current cough management guidelines [19, 20]. Children are managed and investigations performed as clinically indicated.

### Data collection

At enrolment, demographic, epidemiological, cultural, economic and clinical data are recorded including, but not limited to; reason for presentation to the clinical, the presence of any respiratory symptoms, healthcare utilisation prior to presentation, current and past medical history, medication use, treatment and diagnosis, clinical investigation results, socioeconomic status and direct and indirect cost of ARI. Historical variables and those previously described as risk factors for paediatric ARI and lung disease are also collected, including; past respiratory history, familial history, asthma and lung disease, household information (e.g. number of occupants), pregnancy related factors (e.g. gestational age and birth weight), tobacco smoke exposure and cultural factors (e.g. connection to traditional lands and culture). A bilateral anterior nasal swab is collected at enrolment.

For the economic data, both direct and indirect costs to the family, health service provider and employer/community are collected. (Table 1) Time spent by the providers and associated activities will be measured either as resources/inputs used or the time spent by the health care personnel. Where such data may not be available, as is often the case, mainstream data can be adopted using Indigenous templates [21], or expert judgement or explicit framework maybe used. The valuation of the resources used will involve the use of unit costs from the Medicare Benefits Schedule, Pharmaceutical Benefits Scheme and Aboriginal community expert judgements on certain activities [22, 23].

**Table 1:** Direct and indirect cost of ARI

Family	Employers/Community	Healthcare Service
Medication usage	Time spent seeking healthcare	Medication usage
*- includes over-the-counter and prescribed medications*	*- Time off work with pay*	*- includes over-the-counter and prescribed medications*
	*- Time off work with pay lost*	
	*- Time off usual activity*	
Healthcare seeking travel costs	Extra time spent caring for child	Healthcare service utilisation
*- includes ambulance and community transport services*	*- Time off work with pay*	*- diagnostic tests and complementary/alternative therapies*
	*- Time off work with pay lost*	*- distinguishes between public and private, paid and bulk billed services*
	*- Time off usual activity*	
Time spent seeking healthcare	Healthcare seeking travel costs	Healthcare seeking travel costs
*- Time off work with pay*	*- includes ambulance and community transport services*	*- includes ambulance and community transport services*
*- Time off work with pay lost*		
*- Time off usual activity*		
Extra time spent caring for child		
*- Time off work with pay*		
*- Time off work with pay lost*		
*- Time off usual activity*		
Missed childcare/school		
Missed planned activities		
*- child and others*		

Monthly data collection includes: parent(s) or carers of enrolled children being contacted monthly via telephone, email or home visiting for a period of 12 months. Data collection during this time includes parent-collected nasal swabs, ARI events and changes in child characteristics over time.

### Laboratory methods

The nasal swabs are stored at −80 °C at the Queensland Paediatric Infectious Diseases Laboratory, Royal Children’s Hospital, Brisbane. Nasal swabs are thawed and processed in batches for viral and bacterial identification by polymerase chain reaction (PCR) testing as per our previously described methods.

PCR testing for M*. pneumoniae, S.pneumoniae*, Non Typable *Haemophilis influenzae* (NTHi) and *M. cattarhalis* is conducted and a 16S signature sequence to detect all strains of Chlamydiales is used as an initial screen before + ve specimens are tested for specific *C. trachomatis, C pneumoniae* and *S. negevensis* sequences.

PCR will also be used to detect 17 viruses associated with the human respiratory tract including; adenovirus, respiratory syncytial virus, influenza virus types A & B, parainfluenza virus types 1–3, human metapneumovirus, human rhinoviruses, human coronaviruses (OC43, 229E, NL63 + HKU1), human bocavirus and human polyomaviruses KI and WU. Specimen extracts to be tested from each individual will be pooled and will undergo PCR testing for the above-mentioned respiratory pathogens.

### Data handling and storage

Data are collected using paper-based case report forms and entered into a secure Filemaker Pro V12.0 (Filemaker Inc, Santa Clara, CA) database. On completion of study participation, the Chief Investigator reviews all participant files and approves them for sign off and storage. All study folders are stored in a locked secure cabinet accessible only to study staff unless required by legislative or regulatory agencies. Data collected throughout the study are monitored and participant files checked against source documents for completeness and accuracy at completion of each stage.

### Sample size

In the absence of published data on the incidence of respiratory illness, in urban Aboriginal and Torres Strait Islander children at the community level, calculating a sample size on *a priori* evidence is not possible. A study of urban children living in Melbourne during the winter months reported an incidence of influenza-like illnesses of 0.53 episodes per child-month (95 % CI 0.44-0.61) [24]. If we assume a similar incidence, 242 children will be sufficient to detect this rate with a 95 % CI and margin of error of 5.8 %. As the incidence is likely to be higher, a preliminary analysis will be performed when 120 children are enrolled to determine whether the *a priori* sample size can be modified.

### Data analysis

Broadly, descriptive and analytical statistical methods will be utilised including univariate and multivariate logistic and Poisson regression to identify independent predictors of study endpoints. Demographic, clinical, laboratory, socio-economic and risk factor data will be tabulated and expressed as proportions and/or means of the selected characteristics with the corresponding 95 % Confidence Intervals (CI).

Economic analyses will be done according to established methods [25]: including detailed subanalyses of data that account for epidemiological, social, cultural, risk factor and microbiological variables.

The primary analysis will be the incidence density of ARI over a 12-month period. Differences in demographic, clinical, laboratory and risk factor data between children who do and do not develop ARI will be assessed by the normal test for comparisons of means and *χ*^2^ tests for comparison of proportions. The incidence of ARI and the predictors for episodes will be assessed assuming a Poisson distribution and multivariate analyses performed using Poisson regression methods. The denominator will be child weeks of observation, with the number of days of ARI experienced for each child removed from person-time at risk calculations.

To estimate the cost of ARI illness, the costing approach will involve following three steps [26]: a) Identification of the appropriate resources used, b) Measurement of resources used; and c) valuation of such resources used. Identification of the resources or inputs used, such as health care personnel, transport, consumables, will be guided by the ARI clinical or care pathway, which will be used to identify key care activities and associated unit costs. The pathway will reflect the purpose of the exercise, identify all the components or elements of the program/service in a linear manner, and may involve disease progressions [27]. Some or all of the following type of activities will be included in the costing, which may differ from costs in non-ACCHS services: Co-payments for the pharmaceutical products; transport of clients to and from clinic /or services, social and cultural issue that clinic staff with on behalf of client’s e.g. economic hardship, legal issues. The units of measure of costing data will then be combined with the unit costs to estimate the cost of each activity. The costs of the activities will be aggregated to estimate the cost of illness in Indigenous peoples.

## Discussion

To our best of our knowledge, this will be the first Australian study and one of the few worldwide to comprehensively evaluate ARIs and their outcomes in Indigenous children living in an urban area. To date, the focus on ARIs has been almost entirely on children living in rural and remote regions of Australia, with no community based data on disease and its social and economic impacts in urban Indigenous children. Our prospective cohort study design in conjunction with a comprehensive clinical, epidemiological, economic and microbiological collection of data will address issues at both the time of presentation and during the recovery phase of cough illness. Data from this study will likely contribute to future research aiming to develop evidence-based guidelines to improve the early detection, prevention and management of chronic cough in children during and after ARI.

### Rationale for study endpoints

Our definition of ARI that incorporates cough as a symptom is designed to be more specific for events that are likely to lead to higher morbidity and reflect illnesses such as bronchiolitis and pneumonia, important precursors to the development of chronic lung diseases in children, particularly if recurrent [20]. Monitoring the development of chronic cough (i.e. > 4 week’s duration) is therefore a key secondary endpoint given its role as an indicator of lower airways disease. Enrolling children for a period of 12 months and incorporating monthly follow up will enable us to measure changes in predictors (e.g. child care attendance, changes in breastfeeding and vaccination status and exposure to tobacco smoke) and impacts over time.

Our economic endpoints, using a societal perspective, will include cost to the parent/family, cost to the health care sector and other sectors of the economy, such as school attendance and absenteeism from employment [25]. This will enable a complete analysis of the cost of illness that incorporates both direct and indirect costs. Establishing these costs will enable the development of interventions to reduce the burden of disease that incorporate the cost effectiveness of these strategies.

Our microbiological endpoints will provide new information on the characteristics of these infections in urban Indigenous children at the community level given very few children presenting to primary care settings with ARI undergo bacterial and viral testing. The collection of bilateral anterior nasal swabs on all study children will identify viral and bacterial pathogens associated with ARI and cough at both the time of enrolment, and at monthly time points and specialist review. An extended panel of known respiratory pathogens (previously discussed) was chosen to ensure that pathogens excluded from routine respiratory PCR testing were included for analysis and contribute to the literature on pathogens for which the association between nasal detection and clinical illness is still being explored (e.g. polyomaviruses) [28]. Using an extended panel also enhances our ability to explore viral and bacterial interactions and their effects on cough outcomes. However, associating viral detection with current illness is not straight forward as up to 42 % of children who have a non-classical virus detected are asymptomatic [29]. Also, co-detection of viruses and/or virus with bacteria are also commonly found in children with acute and chronic respiratory illnesses [30, 31].

A major strength of our study is that Aboriginal research officers are undertaking all recruitment and data collection in an Indigenous specific primary health care setting, recruitment and data collection is conducted in a culturally safe environment. The relationship between the researcher and participants established with mutual respect and empathy under an ‘indigenous’ umbrella of ‘unwritten’ respect and trust better known as an indigenous methodology, indigenous way of doing [32].

### Limitations

#### Selection bias

A difference may exist between families who seek care through MM and those who attend other primary care facilities. As MM services an area of predominantly low socio-economic status, ARI incidence and its predictors/outcomes may be higher than other settings. It is plausible that parents of children with recurrent ARI are more likely to enrol and complete the study, potentially overestimating the prevalence of chronic cough at day 28. Similarly children with existing undiagnosed chronic lung disease may be enrolled in the study also inflating the prevalence of chronic cough. However, the identification of these children will be an important outcome for the child and his/her family. Our extensive baseline data collection and comprehensive assessment of child at specialist review will allow assessment of these potential biases.

There is also the potential of bias derived from loss-to-follow-up, particularly as those who complete the study may differ from those who do not. However it is expected that this will be limited due to the relationship between the researcher and participants established with mutual respect and empathy under an ‘Indigenous’ umbrella of ‘unwritten’ respect and trust better known as an Indigenous methodology, Indigenous way of doing [32]. Early specialist referral (within 2 weeks of day 28) as part of the study is likely to underestimate costs in children with chronic cough and is therefore, more likely to represent the cost-of-illness incurred with early intervention. However, retrospective collection of cough persistence and healthcare utilisation data at the time of enrolment will allow for assessment of such a bias. The collection of detailed symptom, treatment and clinical data, as well as the collection of study specific anterior nasal swabs will minimise the effect of any potential bias.

#### Measurement bias

Clinician diagnosis may vary between doctors at the clinic due to experience and expertise consequently diagnosis, clinical investigations performed and treatment given may vary between clinicians. Our collection of detailed symptom, treatment and clinical data will facilitate standardising study specific case definitions.

Proxy reporting by parents of cough persistence and type via a diary card using a VCD score was the chosen method for recording cough data during ARI follow up [33]. Even though some misclassification of cough may occur, Chang et al. [34] report that VCD diary cards have the highest correlation to cough frequency when measured objectively and hence, can be used in the absence of practical objective measures to evaluate cough severity and persistence. At specialist review (where required), both the parent and treating physician report on cough type allowing for the assessment of inter-rater reliability and the potential for derived bias Through employing a small team of study specific respiratory specialists from the same centre, the potential for bias derived from inconsistency of diagnosis at the time of specialist review will be minimised.

## Conclusion

ARIs are an inadequately researched cause of morbidity in urban Indigenous children. This study will describe the natural history, epidemiology, aetiology, outcomes and cost of ARI in urban Indigenous children. The results will inform studies for evidence-based guidelines to improve the early detection, prevention and management of chronic cough in children during and after ARI.

## Abbreviations

ARI: Acute respiratory illness
cHRCT: Chest high resolution computerised tomography
CSLD: Chronic suppurative lung disease
NHMRC: National Health and Medical Research Council
PBB: Protracted bacterial bronchitis
PCR: Polymerase chain reaction
VCD: Verbal category descriptive
MM: Murri Medical
RI: Respiratory Illness
SMS: Short message service
